# Circadian rhythm in immunotherapy and cellular therapy: impacts on the tumor microenvironment: Circadian rhythm in immunotherapy

**DOI:** 10.3724/abbs.2025203

**Published:** 2025-11-20

**Authors:** Xiaoyang Sun, Lulu Qin, Xinghua Liang, Dongrui Wang

**Affiliations:** 1 Bone Marrow Transplantation Center of the First Affiliated Hospital and Liangzhu Laboratory Zhejiang University School of Medicine Hangzhou 311121 China; 2 Institute of Hematology Zhejiang University Hangzhou 311121 China; 3 Zhejiang Province Engineering Research Center for Stem Cell and Immunity Therapy Hangzhou 311121 China

**Keywords:** circadian rhythm, immunology, immunotherapy, cell therapy, tumor microenvironment

## Abstract

Immunotherapy, including cellular therapy, has emerged as a crucial pillar in cancer treatment, complementing established modalities such as surgery, chemotherapy and radiotherapy. The clinical observation that immunotherapy is effective in only a limited proportion of patients inspires mechanistic research on the complicated regulatory network within the tumor microenvironment (TME). Circadian regulation significantly affects immune cell behavior, including the activity of immune cells and cytokine production, and emerging evidence suggests the key role of circadian regulation in the TME, which subsequently affects the effectiveness of immunotherapy. Results from preclinical and clinical studies indicate that appropriate timing of adoptive cellular therapy and immune checkpoint blockade therapy improves their efficacy. Therefore, understanding the molecular mechanism of the circadian rhythm together with its role in immunotherapy is essential for optimizing cellular function, proliferation and persistence in the TME. Here, we review how circadian rhythms influence immunotherapy and the TME across different stages of tumor progression. Future clinical protocols may integrate concepts of circadian rhythm and immunotherapy to enhance treatment response.

## Introduction of the Circadian Rhythm

Circadian rhythms are innate cycles that last approximately 24 hours and regulate physiological and behavioral processes. These rhythms are orchestrated by an internal timekeeping system commonly referred to as the biological clock, which aligns bodily functions with the environmental light-dark cycle [1]. Mechanistically, circadian rhythms are regulated by interlocking feedback loops involving genes such as *CLOCK*, *BMAL1*, *PER*, and *CRY* and their protein products, which regulate gene expression and downstream physiological pathways [2]. These loops generate self-sustaining oscillations even under constant environmental conditions, although they can be synchronized by several external cues, particularly light, temperature, and feeding schedules [1]. The circadian rhythm in mammals consists of both central and peripheral rhythms. The central rhythm governed by the suprachiasmatic nucleus (SCN) of the hypothalamus synchronizes the body, whereas peripheral rhythms occur in most tissues and organs, regulating processes such as metabolism, hormone release, and cardiovascular function [3]. Coordinating these clocks ensures temporal order across biological systems, optimizing function and survival.

Circadian rhythms, driven by the transcriptional feedback loops of core clock genes, orchestrate a wide array of key physiological processes, such as cell cycle progression, DNA repair, hormone secretion, and especially immune homeostasis. This interplay, often referred to as chrono-immunology, has become a key area of study for understanding how fluctuations in physiological processes across the diurnal cycle influence immune function. Specifically, circadian rhythms modulate immune responses by affecting the expression of clock genes in macrophages, T cells and other immune cells, leading to differential levels of cytokine release, antigen presentation, and cell migration [4]. A comprehensive review revealed that both innate and adaptive immune responses exhibit rhythmic behavior [5]. For example, the circadian regulation of DC activity and T-cell proliferation results in time-of-day-dependent responses to infection and vaccination efficacy. More recent findings have explored the mitochondrial-level regulation of immune cells by the circadian clock, revealing how oxidative phosphorylation and energy production are also time sensitive and further linking the circadian rhythm to the metabolism of immune cells [6]. In addition, mitochondrial isocitrate dehydrogenase is associated with CAR-T-cell function through histone acetylation and metabolism [7]. Circadian rhythm disruptions alter cellular metabolism, DNA repair, and immune function, which can accelerate aging and cancer development [8]. The circadian rhythm intricately tunes the immune system through gene regulation, hormonal balance, and cellular metabolism. Potential therapeutic strategies, including chronotherapy and anti-aging interventions, are proposed to target these interconnected pathways for improved cancer management [8]. Recognizing this link has opened new avenues in chronotherapy, where treatments such as vaccines or immunomodulators may be optimized on the basis of time-of-day administration [9].

## Disruption of the Circadian Rhythm Promotes Tumor Progression

Circadian rhythm disruption, which is usually caused by shift work, jet lag, or sleep deprivation, can dysregulate a wide variety of physiological functions and lead to pathological conditions, including tumorigenesis. Born *et al*. [10] reported that the quantity and type of circulating immune cells in the blood vary significantly depending on sleep and circadian phase, highlighting sleep as a modulator of immune activity. In addition, disruption of core circadian genes can impair the control of key tumor suppressor pathways. PER2 mutants exhibit elevated rates of spontaneous and radiation-induced tumor formation due to deregulated c-Myc and Cyclin D1 and reduced p53 activity [11]. Melatonin, a circadian system-regulated hormone, has oncostatic effects by inhibiting tumor cell proliferation, inducing apoptosis, and scavenging free radicals. Suppression of melatonin by light at night may eliminate this protective effect, especially in hormone-dependent cancers such as breast cancer [12]. Melatonin was found to upregulate Wnt/β-catenin signaling-related genes in dermal papillae cells, with the effect mediated through increased Wnt ligand expression in hair follicle stem cells, suggesting its potential as a therapeutic agent [13]. In addition, melatonin exerts oncostatic effects by suppressing key pathways involved in prostate cancer progression, including the inhibition of HIF-1α and VEGF for anti-angiogenesis effects and the downregulation of NF-κB to promote apoptosis [14]. Disruptions in circadian rhythms, often caused by modern lifestyles, are linked to cancer development, partly because of their impact on the PI3K/AKT pathway, which regulates cell survival, proliferation, and metabolism [15]. Circadian rhythm disruption affects tumor metabolism by altering glycolysis and lipid metabolism, often leading to metabolic reprogramming that supports cancer cell growth and survival [16].

Chronic circadian disruption can skew the tumor microenvironment (TME) toward an immunosuppressive state, marked by altered cytokine secretion, immune checkpoint expression, and extracellular matrix remodeling, which facilitates tumor progression and immune evasion [ 17, 18] . Circadian misalignment can impair innate and adaptive immune functions, weakening tumor immunosurveillance. Immune cell populations ( *e.g*., cytotoxic T cells and natural killer cells (NK)) exhibit circadian oscillations in function, indicating that immunotherapy administered at the wrong circadian phase can underperform [ 19, 20] . Alterations in circadian clock genes, particularly *CLOCK* and *BMAL1*, can drive metabolic reprogramming in cancer cells and subsequently affect the nutrient supply and metabolic status of immune cells in the TME [21]. Experimental approaches to restore circadian gene expression, such as nanovaccines targeting hypoxia-related circadian disruption, have improved tumor control and enhanced immunotherapy efficacy [22]. Given the significant impact of circadian rhythms on anticancer therapy efficacy, chronotherapy, in which treatment is timed according to the body’s biological rhythms, has emerged as a novel approach to optimize outcomes [23]. Finally, chronic circadian misalignment ( *e.g*., social jet lag or rotating shift work) can predispose individuals to psychiatric conditions and metabolic syndromes by altering stress resilience and neuroendocrine regulation [ 24, 25] . In short, circadian disruption reshapes the TME toward immunosuppression and modulates immunotherapy responsiveness, making clock-aware strategies a promising frontier in cancer treatment.

## Molecular Mechanism of Circadian Rhythm Impacts on TME

The circadian rhythm is an intrinsic time-keeping system that operates on an approximately 24-h cycle and is regulated by transcriptional-translational feedback loops (TTFLs) comprising core clock genes and their proteins. As transcription factors, CLOCK and BMAL1 drive the expression of core circadian genes, including the Period ( *PER1*, *PER2*) and Cryptochrome ( *CRY1*, *CRY2*) genes [26]. Following translation, the PER and CRY proteins assemble into complexes in the cytoplasm, translocate to the nucleus, and inhibit *CLOCK:BMAL1* transcriptional activity, thereby repressing their own transcription. PER and CRY proteins undergo phosphorylation by CK1δ/ε, which targets them for ubiquitin-mediated proteasomal degradation and resetting of the loop [27]. This feedback loop results in rhythmic gene expression over approximately 24 hours. Additional interlocking loops and regulatory elements such as REV-ERBα/β and RORα/γ further refine the robustness and stability of the clock. Below, we briefly summarize how this regulatory network is involved in the formation and evolution of the TME.

### PER1 and PER2

Downregulation of the circadian genes *PER1* and *PER2* has been consistently linked to adverse clinical features across multiple cancer types, including poor tumor differentiation, high Tumor, Node, Metastasis; tumor classification (TNM) staging, increased metastatic potential, and reduced overall survival (OS). Loss of PER2 has been shown to arrest tumor cells at the G2/M phase, linking its circadian role to direct cell cycle checkpoint control, which in turn impacts tumor growth dynamics [28]. In non-small cell lung cancer (NSCLC), aberrant or reduced *PER1* and *PER2* expression significantly correlates with poor differentiation, lymph node metastasis, and increased TNM stages. Importantly, these reduced levels are predictive of poorer patient survival, suggesting their potential as prognostic biomarkers [29]. Similarly, PER2 downregulation is associated with tumor progression, poor differentiation, and enhanced metastasis, reinforcing its role as a tumor suppressor [30]. In gastric cancer, the downregulation of both PER1 and PER2 is related to lymph node metastasis, poor differentiation, and shortened survival, further establishing the roles of these genes in inhibiting cancer aggressiveness [31]. A meta-analysis of several cancer types confirmed these findings broadly: low PER1 and PER2 expression was associated with high-grade tumors, poor differentiation, advanced TNM stage, increased likelihood of metastasis, and significantly reduced OS [32]. Studies in colorectal and oral cancers have drawn similar conclusions. For example, lower PER1 levels were significantly correlated with liver metastasis in patients with colorectal cancer and reduced 5-year survival in patients with oral squamous cell carcinoma [ 33, 34] . PER1 and PER2 are core circadian clock components that are regulated by and modulate the TME. The hypoxic TME, together with inflammatory cytokines and oxidative stress, regulates both PER1 and PER2 via transcriptional feedback loops of the circadian machinery and post-translational modifications [35]. On the other hand, PER1 and PER2 act as molecular links between circadian timing and TME remodeling, with PER2 having a more prominent role in immune and stromal regulation, while both contribute to cancer cell cycle control and tumor-immune interactions. PER2 in particular is essential for fostering a tumor-suppressive TME by promoting collagen deposition and cancer-associated fibroblast (CAF) recruitment [1]. Collectively, these findings underscore the tumor-suppressive roles of PER1 and PER2, linking their downregulation to more aggressive phenotypes and worse prognoses in multiple cancers.

### BMAL1

BMAL1, a core circadian transcription factor, has been recognized for its role in shaping the TME, influencing processes such as immune regulation, metabolic reprogramming, and metastasis. Several studies have demonstrated that BMAL1 regulates immune infiltration and the stromal composition within tumors. For example, BMAL1 disruption promotes the recruitment of immunosuppressive microglia into the glioblastoma TME via CLOCK-BMAL1 signaling, facilitating tumor progression and immune evasion [36]. Similarly, circadian disruption of BMAL1 in mice reshaped the tumor-immune microenvironment to promote tumor cell proliferation by reducing cytotoxic immune activity and altering inflammatory signaling [37]. BMAL1 is also implicated in modulating the physical and metabolic characteristics of the TME. In breast cancer, hypoxia and acidic pH, which are hallmarks of the TME, have been shown to suppress BMAL1 expression, leading to enhanced metastatic potential; conversely, maintaining BMAL1 expression under these conditions may help counteract tumor dissemination [38]. In line with these findings, circadian variations in the TME, such as metabolic flux and vascular activity, correlate with BMAL1 expression levels, suggesting that the timing of therapies to match BMAL1 circadian peaks may optimize efficacy, especially for treatments such as temozolomide in glioblastoma [38]. Moreover, BMAL1 performs tumor-suppressive functions. Knockdown of *BMAL1* in glioblastoma models accelerated tumor growth and modified astrocytic support within the TME, confirming its regulatory effect on non-tumor cellular components [39]. Similarly, BMAL1 deletion activated a metastatic cascade through the PAI-1-TGF-β-myoCAF pathways, increasing fibroblast-mediated support for invasive phenotypes [40]. Collectively, these findings position BMAL1 as a crucial link between circadian rhythms and tumor biology, with a significant influence on both the structural and immune landscape of the TME. BMAL1 orchestrates a molecular cascade involving PAI-1 suppression, activation of TGF-β signaling, and conversion of CAFs into metastasis-promoting myofibroblasts. BMAL1 transcriptionally upregulates plasminogen activator inhibitor-1 (PAI-1), an inhibitor of tissue plasminogen activator (tPA) and urokinase plasminogen activator (uPA). When BMAL1 is disrupted or downregulated, PAI-1 level decreases, leading to increased plasmin activity, which liberates latent TGF-β from its extracellular matrix-bound form into its active signaling state. This increase in active TGF-β stimulates fibroblasts in the TME to acquire a myofibroblastic phenotype characterized by α-SMA expression and increased ECM remodeling capacity, which are hallmarks of metastasis-promoting CAFs [40]. This BMAL1-PAI-1-TGF-β axis has been further validated, showing that BMAL1 deletion in the tumor stroma enhances the transition of CAFs into myofibroblasts and increases tumor invasiveness and metastatic potential [41]. The functional importance of this axis is also underscored by evidence that BMAL1 modulates TGF-β responses across various tissue types, contributing to epithelial-mesenchymal transition (EMT), fibrosis, and cancer progression [42]. Additional work by Riaz *et al*. [43] corroborates that clock disruption intensifies fibrotic stromal reprogramming, further promoting metastatic niches through CAF expansion, collagen deposition, and immune suppression. The circadian clock plays crucial roles in keeping reactive oxygen species (ROS) at safe levels in organisms, minimizing oxidative damage to tissues and cells [44]. Dysregulation of the circadian rhythm may lead to cell stress. NRF2, which is normally degraded by the proteasome, accumulates in the cytosol when circadian rhythm disruption occurs. When BMAL1 is present, the NRF2 complex is translocated into the nucleus, where it activates signals of detoxification, anti-oxidation, metabolism, and anti-inflammatory effects. In comparison, the absence of BMAL1 results in NRF2 accumulation in the cytosol and induces ROS stress in the cell, which is a key factor in tumorigenesis [45]. Thus, BMAL1 functions as a critical gatekeeper linking circadian rhythm integrity to metastatic remodeling of the tumor stroma via the TGF-β signaling pathway.

### CLOCK

Disruption of the circadian rhythm is associated with tumorigenesis, including apoptosis, cell cycle regulation, and DNA damage, which affect tumor staging [46]. CLOCK is a key transcription factor related to circadian rhythm. The upregulation of its expression is associated with increased levels of tumor invasion and proliferation [45]. As a core circadian regulator, the *CLOCK* gene modulates the TME by influencing immune evasion, cellular metabolism, and tumor progression through circadian-mediated gene expression. Research has shown that CLOCK and other circadian components regulate immune infiltration and cellular interactions within the TME. For example, CLOCK was found to actively recruit immune-suppressive microglia in glioblastoma, promoting tumor progression by reshaping the immune microenvironment to favor tumor survival. The depletion of CLOCK in glioma stem-like cells reduces immune suppression and tumorigenicity, underscoring its critical role in TME dynamics [36]. In thoracic cancers, circadian dysregulation, including altered CLOCK expression, remodels the TME by affecting cytokine signaling, angiogenesis, and stromal remodeling [47]. Similarly, in renal clear cell carcinoma, CLOCK and related genes are correlated with immune checkpoint expression and immune cell infiltration, suggesting that circadian components help establish immune-evasive environments [48]. Moreover, CLOCK regulates cancer stem cell (CSC) functions and their niche in the metastatic microenvironment. Disruption of circadian control leads to increased CSC plasticity, invasion, and resistance to therapy, implicating CLOCK in the orchestration of both the tumor architecture and metastatic competence [1]. A review of circadian clocks in the broader oncological context emphasized how the rhythmic expression of CLOCK influences angiogenic factors, extracellular matrix composition, and metabolic crosstalk between tumors and stroma, reinforcing its essential role in TME regulation [35]. Finally, analyses across tumor types highlight that spatiotemporal variations in CLOCK expression within the TME shape treatment response and tumor aggression, with implications for circadian-based interventions [19]. In summary, CLOCK not only serves as a cellular timekeeper but also actively governs the TME composition, immune dynamics, and therapeutic response, making it a promising target for chrono-immunotherapy.

### CRY1/2

The core circadian genes *CRY1* and *CRY2*, encoding cryptochromes, have several correlations with the TME, influencing tumor progression, immune responses, and therapy resistance. CRY1 expression is frequently upregulated in various tumors and is linked to more aggressive phenotypes and therapeutic resistance. In renal and thoracic cancers, CRY1 is associated with immunosuppressive TMEs and resistance to drugs, indicating its potential role in dampening anti-tumor immunity [ 47, 48] . Specifically, a pancancer study in 2020 revealed that high CRY1 expression is positively correlated with immune exclusion and resistance signatures, suggesting its influence on immune evasion [49]. Mechanistically, CRY1 can alter DNA repair rhythms, creating a survival advantage for tumor cells under genotoxic stress, thereby reinforcing a hostile TME [50]. CRY2, while less rigorously studied, appears to have distinct, sometimes opposing functions. In breast cancer, CRY2 is epigenetically downregulated in more aggressive subtypes, suggesting that it has tumor-suppressive properties [51]. Similarly, in prostate and colorectal cancers, reduced CRY2 expression is associated with immune evasion and increased malignancy [52]. Interestingly, the dual expression of CRY1 and CRY2 may influence cancer prognosis and immune infiltration patterns. In 2022, a study on lung adenocarcinoma identified CRY1 and CRY2 as part of a circadian gene signature with predictive value for immune checkpoint therapy responsiveness [53]. In summary, CRY1 tends to promote oncogenic features and immunosuppression within the TME, whereas CRY2 may act more like a tumor suppressor, particularly in hormone-sensitive or colorectal cancers. These opposing roles suggest that therapeutically targeting cryptochrome signaling could modulate the TME and improve responses to immunotherapies or chronochemotherapies.

### NR1D1/NR1D2

The nuclear receptors NR1D1 (Rev-erbα) and NR1D2 (Rev-erbβ), regulators of the central circadian rhythm, play significant and nuanced roles in the TME, affecting immune infiltration, inflammation, and cancer progression. In lung cancer, Kim *et al*. [54] reported that NR1D1 deficiency promotes tumor development through activation of the NLRP3 inflammasome within the TME. This pro-inflammatory environment facilitates cancer progression, highlighting the role of NR1D1 as a tumor suppressor. Similarly, NR1D2 was found to perform tumor-suppressive functions in lung cancer, possibly through overlapping pathways, although the exact mechanisms involved remain under investigation. In breast cancer, NR1D1 activates the cGAS-STING signaling pathway, promoting antitumor immune responses [55]. Notably, manganese sensitizes the cGAS-STING pathway by increasing cGAS enzymatic activity and STING-binding affinity, even at low cytoplasmic DNA levels, making it a potent adjuvant for cancer immunotherapy [56]. These results suggest that NR1D1 is a positive regulator of innate immune activation within the TME, counteracting immune evasion mechanisms in cancer cells. NR1D2 also plays a critical role in glioblastoma, where Yu *et al*. [57] discovered that it regulates both cell proliferation and motility, potentially affecting how tumor cells interact with and remodel the TME. Notably, its knockdown resulted in altered expression of polymerization-related genes and AXL, a receptor tyrosine kinase linked to immune suppression, which further highlights the immunomodulatory roles of AXL in shaping TME dynamics. In summary, NR1D1 and NR1D2 function not only as circadian regulators but also as pivotal regulators of the TME through immune modulation, inflammasome regulation, and tumor suppressive signaling. Their expression profiles may serve as prognostic indicators and potential therapeutic targets across multiple cancer types.

### RORγ

Retinoic acid-related orphan receptor gamma (RORγ), a nuclear receptor that acts as a transcription factor, modulates both circadian rhythm and immune responses. RORγ, along with its isoform RORα, is a critical activator within the transcriptional-translational feedback loop (TTFL) of the circadian rhythm. These RORs bind to the promoter regions of core clock genes, notably *BMAL1*, thereby sustaining circadian oscillations. Disruption of RORγ expression alters the timing and amplitude of these oscillations, with downstream effects on metabolic, hormonal, and immune system regulation [35]. The circadian clock, via components such as RORγ, also influences the TME. RORγ has immunomodulatory effects, especially in promoting Th17 differentiation and cytokine expression. In cancer contexts, the circadian activity of RORγ impacts immune cell infiltration, antigen presentation, and cytokine rhythms, all of which are crucial to tumor immune surveillance [58]. Recent studies have shown that the dysregulation of ROR family members, including RORγ, is correlated with reduced expression of immune checkpoint targets such as PD-L1 in tumors, enhancing antitumor immunity. Specifically, RORα (a close homolog) has been shown to suppress PD-L1 expression, suggesting that RORγ might have similar effects [59]. Furthermore, circadian rhythm disruption in tumors fosters a pro-inflammatory TME, facilitates EMT, and weakens antitumor immunity, making clock-regulating proteins such as RORγ viable therapeutic targets for chrono-immunotherapies [ 60, 61] . In glioblastoma and other cancers, emerging evidence links circadian clock dysregulation, potentially through altered RORγ signaling, with cancer stem cell maintenance, tumor aggressiveness, and therapy resistance [1]. Thus, RORγ is a nexus point connecting circadian biology and tumor immunology, offering potential for both prognostic biomarkers and therapeutic intervention.

## Effects of Circadian Rhythm on Non-Immune Cells in the TME

### Cancer stem cells

**Figure 1:**
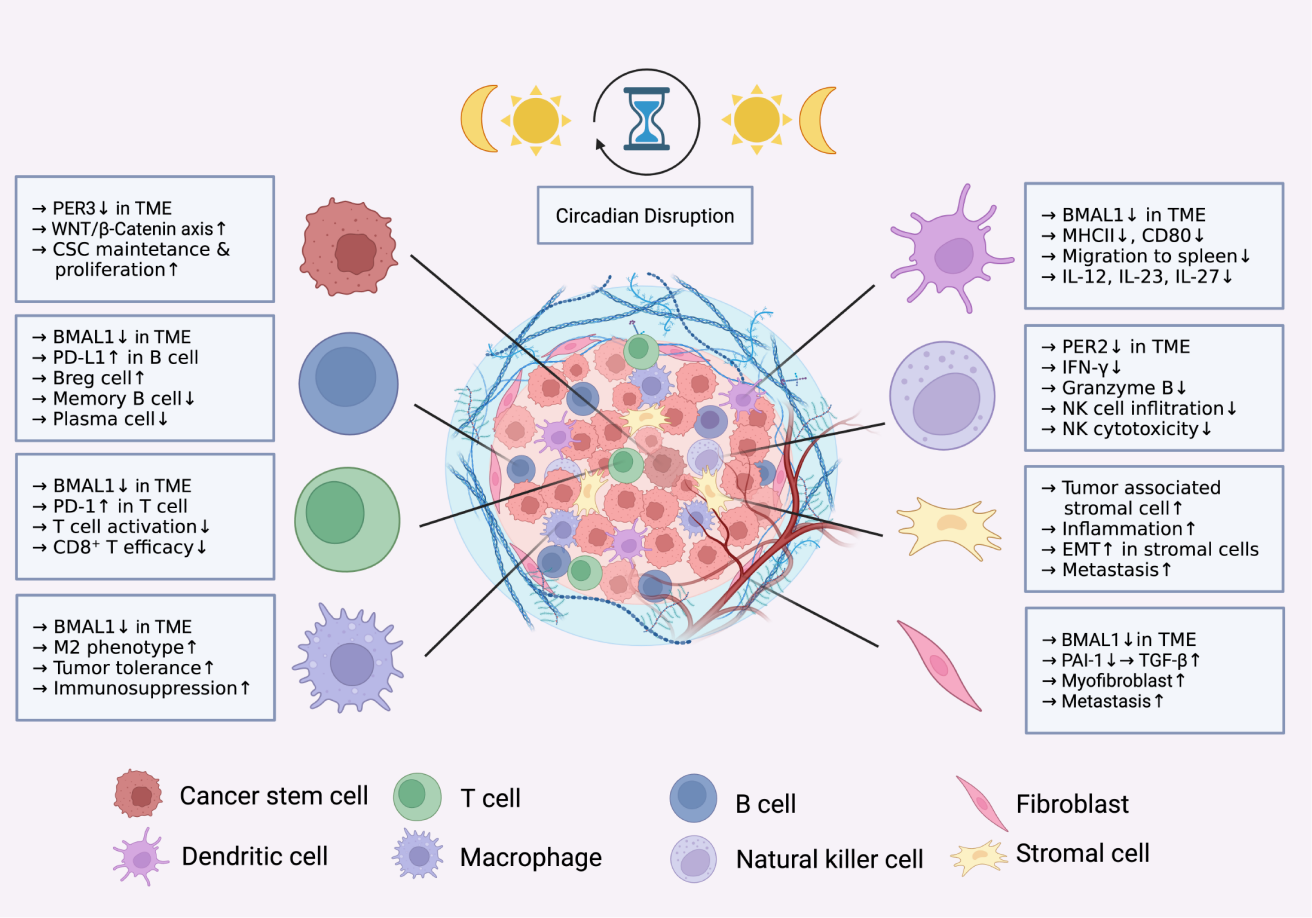
Figure 1 Impact of circadian disruption on immune cells and tumor-associated cells in the TME The schematic shows how circadian disruption promotes tumor progression through dysregulation of circadian genes such as BMAL1, PER2, and PER3, with downstream effects on these pathways.

The circadian rhythm affects the behavior of cancer stem cells (CSCs), which is associated with tumor progression, metastasis, the immune response, and treatment responsiveness. Recent studies have revealed that circadian regulation affects CSC plasticity and metastatic potential. For example, the circadian transcription factor BMAL1 promotes metastatic outgrowth by upregulating genes that support a pro-metastatic TME for the seeding of CSCs, suggesting temporal control of metastasis by core clock components [1]. In prostate cancer, the circadian clock gene *PER3* was found to negatively regulate CSC stemness by inhibiting the WNT/β-catenin signaling pathway, a key axis in CSC maintenance and proliferation [62]. The role of circadian disruption has also been implicated in enhancing CSC traits. A pivotal mouse model study demonstrated that chronic circadian disruption enhanced breast CSC properties and reprogrammed the immune microenvironment to a pro-metastatic state, especially through increased expression of inflammatory cytokines and immune evasion markers [63]. Further insights from analyses of the EMT process, which is involved in CSC generation, suggest that this process is temporally regulated by the intrinsic circadian clock [60]. These findings suggest that circadian timing can dictate when tumor cells acquire stem-like, migratory characteristics [64]. Mechanistically, circadian regulators such as CRY1 and CRY2 are being explored as therapeutic targets for CSCs, with compounds such as KL001 showing efficacy in inhibiting glioblastoma CSC growth by activating these repressors and modulating TME dynamics [19]. Finally, chronic chemotherapy not only affects CSC killing but also influences immune cell dynamics within the TME, further enhancing treatment effectiveness. The timing of treatment, such as chronotherapy, which is based on the circadian dynamics of CSC populations, has demonstrated improved efficacy, suggesting that circadian control is vital not only in tumor biology but also in optimizing therapeutic interventions [65]. For example, the administration of aldehyde dehydrogenase inhibitors at times when CSC activity was highest resulted in greater reductions in CSC frequency, suppressed tumor growth, and reduced lung metastasis. In contrast, giving DEAB when the CSC activity was lowest had weaker effects [65]. These studies collectively underscore the importance of circadian biology in modulating the TME and CSC behavior, paving the way for circadian-based therapeutic strategies ( Figure 1).

### Stromal cells

Circadian rhythms exert a profound regulatory influence on stromal cells within the TME, reshaping tumor dynamics and therapeutic responses. These 24-h biological cycles orchestrate transcriptional, metabolic, and signaling programs in both cancer cells and the surrounding stromal milieu. Core circadian clock components modulate stromal cell behavior, affecting CSC maintenance and metastatic potential. For example, stromal cell-specific circadian oscillations influence temporal genetic expression related to the ECM, angiogenesis, and immune modulation, thus reshaping the TME to favor cancer progression [1]. In colon cancer, bidirectional communication between cancer cells and tumor-associated stromal cells alters circadian gene expression, which in turn enhances malignant phenotypes such as invasion and chemoresistance [66]. This interaction disrupts homeostatic rhythmicity, contributing to tumor-promoting inflammation and fibrosis. Studies in glioma and NSCLC highlight that circadian gene dysregulation in stromal compartments is related to poor prognosis and altered infiltration of immune cells [67]. Temporal disorganization in these stromal populations may compromise immune surveillance and disrupt the recruitment of tumor-suppressive immune subsets. Moreover, circadian disruption through genetic or environmental perturbation modulates EMT in stromal cells, which is critical in metastasis and resistance to immune attack [60]. For example, circadian clock loss in stromal fibroblasts has been implicated in enhancing cancer cell invasion and suppressing antitumor immunity. Importantly, these circadian influences extend to shaping the immune landscape of the TME. Circadian disruption alters T-cell infiltration and function, which is mediated partly through stroma-derived cues, leading to an immunosuppressive microenvironment [58]. Together, these findings emphasize that the tumor stroma is not only passive scaffolding but also an activated, time-sensitive participant in cancer biology. Targeting circadian rhythms in stromal cells offers a promising avenue for temporally optimized therapies and chronotherapy strategies [ 35, 38] .

### Non-cell stromal components

The circadian rhythm, a fundamental biological timing mechanism, substantially influences the TME, particularly its noncellular stromal components, such as the ECM, adipose tissue, and secreted signaling molecules. Recent reports have revealed that the circadian clock can regulate structural ECM components, such as collagen and fibronectin, as well as matrix metalloproteinases (MMPs), which are critical for tissue remodeling and cancer cell invasion. Disruption of circadian genes alters ECM dynamics, which can facilitate the growth and metastasis of tumor cells by weakening tissue barriers and promoting cell migration [38]. Moreover, circadian dysfunction contributes to dysregulated adipogenesis within the TME. Adipocytes, particularly in cancer-associated adipose tissue, are subject to circadian control over lipid metabolism, hormone secretion, and inflammatory signaling. Misalignment of circadian signals leads to metabolic reprogramming in both stromal and cancer cells, promoting a pro-tumorigenic environment [ 68, 69] . In the context of brain tumors such as glioblastoma, interactions between tumor cells and non-cellular stromal components, including proteoglycans and ECM-derived signals, are temporally regulated. Circadian disruption modifies these interactions and influences blood‒brain barrier permeability and tumor invasiveness [70]. Importantly, bone marrow adipose tissue (BMAT) and its stromal progenitors, such as mesenchymal stem cells (MSCs), also exhibit circadian oscillations. Crosstalk dysregulation of the aryl hydrocarbon receptor (AhR) and circadian clock components in BMAT has been implicated in hematologic malignancies such as leukemia, where non-cellular stromal support becomes maladaptive [71]. Overall, the circadian rhythm orchestrates the complex regulation of non-cellular stromal components within the TME. Disruptions in this timing system can drive tumor progression by altering ECM integrity, metabolic signaling in the adipose stroma, and the spatial organization of the tumor niche.

## Effects of Circadian Rhythm on TME-associated Immune Cells

### Macrophages

Immune responses in the TME are also under circadian rhythm control. Tumor-associated macrophages (TAM), key players in the TME, exhibit significant temporal regulation that affects tumor progression, angiogenesis, and immunosuppression. Circadian dysregulation in macrophages can promote a pro-tumoral phenotype. Acidic conditions within tumors disrupt circadian oscillations in TAMs, leading to alterations in phagocytosis, cytokine release, and polarization toward an M2-like (pro-tumorigenic) state. For example, Knudsen-Clark *et al*. [72] reported that acidic TME conditions alter macrophage circadian gene expression, promoting tumor tolerance. Additionally, circadian clocks modulate the M1/M2 polarization axis. The proper circadian rhythm favors M1-like (anti-tumoral) polarization, whereas disruptions skew toward M2 phenotypes, reducing the immune system’s capacity to combat tumors [38]. Similarly, Xuan *et al*. [35] described how tumor cell clock genes can influence innate myeloid cells, including macrophages, via exosomal communication and cytokine gradients. In addition to phenotypic shifts, circadian regulation influences macrophage metabolism, phagocytic function, and immune checkpoint interactions. For example, circadian disruption through BMAL1 deletion reduces the inflammatory capacity of TAMs and impairs tumor immune surveillance, as demonstrated by Clark & Altman [73]. Notably, therapeutic strategies targeting the circadian machinery in macrophages are emerging. Yoshida *et al*. [74] proposed that synchronizing macrophage clocks via microcurrent stimulation enhances their phagocytic anti-tumor capacity. Circadian regulators such as BMAL1, CLOCK, and PER1/2 orchestrate oscillations in immune cell behavior, influencing both the differentiation and function of immune cells. Specifically, BMAL1 has been shown to affect macrophage polarization, metabolism, and cytokine production, contributing to TAM phenotype shifts in tumors with disrupted rhythms [58]. The CXCL5-CXCR2 axis plays a critical role in recruiting myeloid-derived suppressor cells (MDSCs) and TAMs into the TME. In chronic circadian disruption models, such as night shift simulations, CXCL5 expression increases in the tumor milieu, leading to enhanced CXCR2-mediated recruitment of MDSCs and TAMs, thus creating an immunosuppressive niche favorable for metastasis [63]. This axis acts rhythmically, as CXCL5 and CXCR2 display circadian fluctuations that align with peak immune suppression and tumor growth phases [1]. Pharmacological inhibition of CXCR2 via antagonists, such as SB225002, disrupts this recruitment pathway, reducing MDSC infiltration and increasing antitumor immune responses, suggesting potential therapeutic avenues [75]. Moreover, circadian disruption affects not only the recruitment but also the functions of MDSCs and TAMs. These cells become more immunosuppressive under altered light/dark cycles, increasing the expression of PD-L1 and producing suppressive mediators such as arginase-1 and IL-10, which are driven in part by increased CXCL5-CXCR2 signaling [63]. These studies underscore how circadian rhythms regulate the immunosuppressive TME by modulating chemokine signaling pathways such as CXCL5-CXCR2, which in turn govern the recruitment and functional polarization of TAMs and MDSCs. Targeting this rhythm-sensitive axis could provide novel chronotherapeutic strategies against cancer. Collectively, the functions of macrophages are regulated by the circadian clock in the TME. Modulating circadian timing may thus offer novel immunotherapeutic strategies aimed at reprogramming macrophages to a tumor-fighting phenotype.

### Dendritic cells

Alterations in the function of dendritic cells (DCs) under circadian rhythm regulation play crucial roles in the remodeling of the immune response in the TME. The antigen presentation and cytokine secretion of DCs are regulated by circadian oscillation, thereby directly influencing their antitumor effectiveness. Wang *et al*. [76] demonstrated that DCs possess intrinsic circadian machinery that governs the timing and amplitude of their immune activation against tumors. The synchronization of DC circadian rhythms with external cues significantly enhanced anti-tumor responses in both murine and human models. Circadian rhythm deregulation, either through genetic or environmental elements such as light exposure, has been shown to impair DC maturation and reduce DC recruitment and activation capacity in the TME. These factors lead to increased tumor growth, as seen in the work of Roberts and MacDonald [77], who reported that circadian disruption in mice elevated the tumor burden and favored the accumulation of myeloid-derived suppressor cells over immunostimulatory DCs. Further research has focused on these findings by linking circadian control of immune checkpoints and cellular recruitment to broader oncological outcomes [ 58, 78] . In NSCLC, circadian gene expression profiles are correlated with reduced DC and M2 macrophage infiltration in tumor tissues, highlighting the broader immune suppression effects of clock gene dysregulation [78]. At the mechanistic level, Zeng *et al*. [58] reported that circadian rhythm genes regulate the expression of cytokines and costimulatory molecules in DCs, directly influencing their maturation and ability to stimulate T cells. The loss of rhythmic expression of these genes compromises DC functionality and impairs adaptive immunity within the TME. In addition, its synchronization enhances immune surveillance and anti-tumor immunity, while its disruption supports tumor progression by impairing DC-mediated immune responses. This insight has strong implications for optimizing cancer immunotherapies on the basis of time-of-day administration strategies.

### NK cells

The circadian rhythm also profoundly influences natural killer (NK) cells in the TME. Hypoxia, which is mediated by hypoxia-inducible factors, promotes tumor immune evasion by downregulating NK cell-activating receptors and inducing resistance in tumor cells through mechanisms such as reduced MICA/B expression and PD-L1 upregulation [79]. Hypoxia also directly suppresses NK cell cytotoxicity by reducing the secretion of cytokines such as IFN-γ and degranulation markers while indirectly inhibiting NK cells via immunosuppressive cells, including Tregs and MDSCs, with metabolites such as adenosine and lactate [79]. Disruption of the circadian rhythm has been increasingly linked to immunosuppressive conditions that favor tumor progression. The circadian clocks modulate the gene expression and cytolytic activity of NK cells at the cellular level. For example, circadian disruption is associated with impaired NK cell function and reduced cytotoxicity, weakening immune surveillance against tumors [58]. Specifically, circadian gene expression, such as BMAL1 and PER2, is directly related to NK cell activation markers, such as IFN-γ and granzyme B. Chronic jet lag models in mice, which mimic circadian disruption, demonstrate decreased NK cell infiltration and functionality in melanoma models, facilitating tumor progression [80]. This links circadian misalignment not only to immune suppression but also to structural alterations in the TME that deter NK cell-mediated cytotoxicity. In another rodent model, tumors showed differential NK cell responses in the spleen depending on the circadian phase, with peak NK activity aligning with host circadian peaks, highlighting temporal windows of optimized immune defense [37]. These effects reflect how peripheral and central circadian regulators synchronize with immune cell trafficking and function. Furthermore, in NSCLC and prostate cancer, gene expression related to the circadian rhythm is associated with differential NK cell abundance and prognosis, suggesting translational implications for circadian-informed therapies [ 52, 78] . Overall, the circadian rhythm orchestrates key facets of NK cell physiology, from transcriptional regulation to tumor homing, making its preservation crucial for maintaining immune competence in the TME.

### T cells

The circadian rhythm regulates T cell biology, which is correlated with disease progression and therapeutic outcomes. The infusion of stem cells early in the day significantly reduces the incidence and severity of graft- *versus*-host disease (GVHD), likely by modulating circadian cytokine responses such as IL-1α and donor T-cell activation [81]. Within the TME, the circadian rhythm has been shown to modulate both the immune surveillance and anti-tumor responses of T cells. Several recent studies have highlighted how circadian oscillations govern T cell infiltration, function, and interaction with other immune cells, ultimately shaping tumor progression and the response to therapy. One central finding is that circadian disruption leads to immune remodeling in the TME, reducing CD8⁺ T-cell efficacy while increasing the proportion of immunosuppressive myeloid cells, thus favoring tumor growth [ 63, 82] . This phenomenon has been documented in murine models, where circadian rhythm disruption led to increased tumor proliferation and accumulation of myeloid-derived suppressor cells [77]. Further evidence suggests that CD8⁺ T cells exhibit rhythmic infiltration into tumors, with their effector functions varying on the basis of time-of-day cues. These oscillations influence immunotherapy outcomes, implying that timing treatments to align with circadian peaks may enhance therapeutic efficacy [82]. The molecular basis for these rhythms involves intrinsic clock genes within T cells. These genes regulate cytokine production, metabolic pathways, and cytotoxic activity, and their dysregulation can impair tumor-specific immune responses [ 58, 83] . Moreover, circadian disruption within cancer cells themselves alters the TME in ways that suppress T cell activation and favor immune evasion. Tumor cells modulate their own clocks and release signals that misalign local immune cell rhythms, thereby dampening cytotoxic responses [ 19, 35] . Finally, studies in lung and endocrine cancers underscore that polarization, abundance, and activation status, including CD4⁺ and CD8⁺ T cell subsets, are temporally regulated in the TME, reinforcing the systemic nature of circadian control over tumor–immune dynamics [ 60, 78] . In summary, T-cell-mediated immunity is strictly regulated by the circadian clock in the TME, with major implications for immunotherapy design and timing.

### B cells

Circadian rhythms profoundly shape immune cell behavior, including the activity and function of B cells within the TME, with emerging studies revealing how rhythm disruptions influence tumor immunity and progression. A foundational review by Xuan *et al*. [35] underscores how circadian regulation modulates both tumor cells and various components in the TME, including immune infiltrates. Temporal control affects not only the proliferation of tumor cells but also the recruitment and polarization of immune cells. In particular, the circadian rhythm can regulate B-cell maturation, antigen presentation, and antibody production. In colorectal cancer models, disruption of the core clock gene BMAL1 led to reduced populations of PD-L1⁺ intraepithelial B cells and altered immune cell interactions, including increased CD4⁺ T cell apoptosis, thereby contributing to tumor progression [84]. Additional evidence from Zeng *et al*. [58] highlights how circadian disruption alters the function of B lymphocytes, although mechanistic insights remain underdeveloped. This work suggests that B cells might contribute to circadian-controlled tumor immune surveillance or suppression depending on the rhythmic context of the cytokine environment and stromal cues. In NSCLC, circadian rhythm gene expression promotes the infiltration of naïve B cells and plasma cells, indicating that the B-cell distribution within tumors may be temporally regulated and carry prognostic relevance [78]. Furthermore, in breast cancer, circadian subtypes are linked to differential immune landscapes, with one subtype exhibiting enriched B-cell-related gene signatures and a better response to immunotherapy [85]. Collectively, these findings indicate that the circadian rhythm shapes both the quantity and functional phenotype of B cells in tumors, influencing immune dynamics and therapeutic responsiveness. Future therapies might harness these insights to synchronize immunotherapies with patients’ biological clocks.

## Exploiting Circadian Rhythm to Enhance the Therapeutic Effect of Immunotherapy

### Immune checkpoint blockade

Immune checkpoint blockade (ICB) therapy works by targeting T-cell-negative regulatory pathways to enhance antitumor immune responses, primarily through the inhibition of checkpoint receptors such as cytotoxic T-lymphocyte-associated antigen 4 (CTLA-4) and programmed cell death protein 1 (PD-1). Under normal conditions, immune checkpoints function as inhibitory signals that suppress T-cell hyperactivation, helping to maintain self-tolerance and prevent autoimmune reactions [ 86, 87] . In the TME, however, these pathways are often exploited by tumor cells to escape immune recognition. For example, PD-1 engagement by PD-L1 or PD-L2, which are expressed on tumor or stromal cells, dampens T cell activation and killing activity. In an ICB phase III trial, pembrolizumab, an immune checkpoint inhibitor drug, combined with concurrent chemoradiotherapy (CCRT) followed by maintenance pembrolizumab significantly improved progression-free survival (PFS) and demonstrated a positive trend in OS compared with CCRT with placebo in patients with newly diagnosed, high-risk, locally advanced cervical cancer [88]. Similarly, CTLA-4 modulates T cell responses by binding to CD80/CD86 on antigen-presenting cells, thereby blocking the CD28-mediated costimulatory signals needed for T-cell activation [89]. In another ICB phase III trial, first-line treatment with nivolumab plus ipilimumab, both of which are immune checkpoint inhibitor drugs, combined with two cycles of chemotherapy significantly improved OS compared with standard chemotherapy alone in patients with advanced NSCLC, providing a clinically meaningful benefit across various patient subgroups [90]. Therapeutic antibodies targeting CTLA-4 (ipilimumab) or PD-1/PD-L1 (nivolumab, pembrolizumab, atezolizumab) prevent checkpoint-mediated inhibition, allowing T cells to recognize and eliminate tumor cells ( Table 1). ICB therapy has transformed cancer treatment by reactivating anti-tumor immunity, yet its efficacy remains inconsistent across patients and cancer types. The diversity of immune reactivity and TME composition present significant clinical challenges, driving inconsistent treatment responses and multifaceted resistance [98]. Additionally, immune-related adverse events and difficulty in determining optimal treatment windows pose critical obstacles to their widespread effectiveness [99]. The antitumor efficacy of these inhibitors depends on various factors, including the immunogenicity of the tumor, the presence of infiltrating lymphocytes, and the expression of checkpoint molecules [ 100, 101] . Despite their success, not all patients respond, and resistance mechanisms, including the lack of high-quality tumor neoantigens, mutations in interferon signaling pathways, the overexpression of alternative checkpoints such as TIM-3 or LAG-3, and immunosuppressive TMEs, have been identified [ 102, 103] . ICB can also be integrated with other therapeutic strategies, such as targeted therapies or CAR-T cells, to enhance efficacy and overcome resistance [104]. This underscores the need for predictive biomarkers and combination regimens tailored to tumor-specific immune landscapes [105].

**Table:** **Table 1** Commercialized ICB products

Drug	Target	Tumor	Approval	Ref.
Ipilimumab	CTLA-4	Stage III or IV malignant melanoma	August 2010	[91]
Tremelimumab	CTLA-4	Unresectable hepatocellular carcinoma	October 2022	[92]
Nivolumab	PD-1	Stage III-B or IV squamous NSCLC	March 2015	[93]
Pembrolizumab	PD-1	Stage IV nonsquamous and squamous NSCLC	October 2016	[94]
Atezolizumab	PD-L1	Stage III-B or IV nonsquamous and squamous NSCLC	October 2016	[95]
Avelumab	PD-L1	Metastatic Merkel cell carcinoma	March 2017	[96]
Durvalumab	PD-L1	Stage III NSCLC	February 2016	[96]
Cemiplimab	PD-1	Metastatic cutaneous squamous cell carcinoma	September 2018	[97]

Emerging evidence suggests that the circadian rhythm can modulate antitumor immune function and ICB responsiveness. For example, circadian misalignment is linked to disrupted immune responses, tumor progression, and reduced efficacy of ICB immunotherapies [20]. Implementing chronotherapeutic approaches that align ICB dosing with physiological rhythms has proven effective for boosting antitumor responses. Treatment outcomes are markedly improved when ICB dosing schedules are synchronized with circadian-regulated immune processes, particularly T-cell responsiveness and inflammatory signaling [ 82, 106] . CD8 ^+^ T-cell infiltration and effector function are circadian-gated, where tumor-infiltrating CD8 ^+^ T cells peak in number and exhibit cytotoxic activity during the daytime in humans [82]. Administering ICB when T-cell activity is naturally elevated enhances tumor control, whereas administering treatment during the circadian rhythm leads to poorer responses. Moreover, both tumor cells and immune cells exhibit circadian-controlled gene expression. Disruption of the circadian machinery of tumor cells impairs immune surveillance and T-cell infiltration, reducing the effectiveness of ICB [ 18, 107] . Restoring or leveraging circadian rhythms could reprogram the TME to favor immune cell recruitment and function. Time-of-day-dependent PD-L1 expression profiles reveal chronotherapeutic opportunities to enhance checkpoint inhibitor efficacy through biologically timed administration [ 108, 109] . Preclinical studies have demonstrated that PD-1 and PD-L1 levels fluctuate with circadian cycles and that blockade of the PD-1/PD-L1 axis is significantly more effective when it is administered during the phase with the lowest PD-1 expression [ 82, 110] . For example, in murine melanoma models, PD-1 expression by TAMs increases at specific Zeitgeber times, with immunotherapy efficacy maximized when treatment coincides with peaks in PD-1 levels, reducing immune evasion [ 106, 111] . In humans, more CD8 ^+^ T cells are exhausted in the afternoon than in the morning, which makes ICBs more effective in the morning [82]. Mechanistically, this rhythmicity is linked to clock genes such as BMAL1 and PER2, which modulate cytokine release, T cell infiltration, and tumor antigen presentation [112]. Sleep and lifestyle disruptions, which are common in cancer patients, can dampen circadian signals, potentially limiting the effectiveness of ICBs. Interventions to restore circadian rhythms, including light therapy, melatonin supplementation, and sleep hygiene, are being explored to synergize with immunotherapy [113]. These findings position chrono-immunotherapy as a promising clinical paradigm in which the timing of checkpoint inhibitor administration could be tailored to individual circadian rhythms for improved therapeutic benefit. Additionally, ongoing research is exploring how to systematically apply circadian-informed scheduling to optimize outcomes and minimize side effects [114].

The timing of ICB administration can be determined by the oscillatory expression of immune-related genes and the dynamics of immune cells. Oscillatory expression of immune-related genes and cells is closely linked to circadian rhythms and intracellular signaling dynamics. These oscillations are critical for shaping immune cell behavior, pathogen defense, and tissue homeostasis. At the systems level, immune processes show rhythmic fluctuations throughout the day, driven by both cell-intrinsic clocks and systemic cues. For example, the number of lymphocytes and their adaptive responses are governed by circadian clocks within T cells, where disruption of these oscillations impairs immunity and worsens the condition of experimental autoimmune encephalomyelitis [115]. Similarly, adrenergic receptor signaling entrains oscillations in circadian genes and effector molecules in CD8 ^+^ T cells, optimizing antiviral responses [116]. At the molecular scale, oscillatory transcription factors play a central role. For example, NF-κB, a master regulator of inflammation and tumor progression, undergoes rhythmic nuclear translocation and spiky oscillations that tune the gene expression outputs necessary for B-cell activation and adaptive responses [117]. Oscillations in immune cells and genes ensure that immune responses are temporally coordinated with environmental cues, such as light/dark cycles and pathogen exposure. This rhythmicity underlies what has been termed “immunity around the clock,” where circadian clocks govern both innate and adaptive immunity [118]. Fortin *et al*. [112] reported that circadian gating of tumor immunosuppression shapes ICB responses, highlighting oscillatory changes in chemokine expression ( *e.g*., Cxcl5) and immune cell activity. Circadian transcription factors directly gate tumor immunosuppression, thereby modulating the effectiveness of ICB. This regulation is tied to the rhythmic expression of chemokines and immune modulators. The BMAL1-CLOCK complex drives circadian oscillations in macrophages, affecting PD-1/PD-L1 pathway activity in sepsis, suggesting that checkpoint pathways are under direct circadian transcriptional control [119]. In addition, PER and CRY clock genes oscillate in immune cells and tumors; disruption of these rhythms diminishes immune surveillance and alters checkpoint-related gene expression [58]. In short, immune oscillations relevant to ICB are largely driven by canonical circadian transcription factors through the rhythmic transcription of checkpoint and immune regulatory genes. Together, these studies point to a “chrono-immunotherapy” paradigm, where aligning ICB administration with the patient’s circadian immune rhythms could maximize antitumor efficacy and minimize immune exhaustion.

### Adoptive cellular therapy (ACT)

ACT represents an immunotherapeutic approach in which immune effector cells are isolated, biologically modified or expanded *ex vivo*, and subsequently reinfused to mediate targeted disease clearance. The fundamental mechanism of ACT hinges on enhancing the body’s own immune system by expanding or engineering T cells with heightened specificity and potency against pathological targets. Therapeutic T cell procurement represents the critical first step, where lymphocyte subsets with optimal expansion potential and effector function are selected from either the patient′s immune repertoire or donor lymphocyte pools. These cells are then either selected for their inherent tumor reactivity ( *e.g*., tumor-infiltrating lymphocytes or TILs) or genetically modified to express receptors such as chimeric antigen receptors (CARs) or engineered T-cell receptors (TCRs): TIL therapy leverages naturally tumor-reactive lymphocytes from tumor biopsies; CAR-T-cell therapy exploits engineered T cells with synthetic receptors that bind specific antigens on cancer cells; and TCR gene therapy introduces T-cell receptors that recognize tumor-associated antigens presented by MHC molecules [120]. Once expanded to sufficient numbers, these enhanced T cells are reinfused into the patient to target and eliminate tumor cells or infected cells with improved efficacy [121]. Additionally, research has demonstrated that multiengineered induced pluripotent stem cell-derived CAR-NK cells targeting CD70 exhibit strong anti-tumor activity and can eliminate alloreactive T cells, suggesting a promising universal immunotherapy platform [122]. The effectiveness of ACT is influenced by various mechanisms, such as T-cell persistence, trafficking to tumor sites, and overcoming immunosuppressive TMEs. Modifications such as the inclusion of suicide genes, the use of small-molecule proteolysis-targeting chimeras (PROTACs), and checkpoint blockade can further improve the safety and function of these therapies [123]. Tumor immune evasion presents a significant challenge, often through downregulation of antigen presentation, secretion of inhibitory cytokines, or recruitment of suppressive immune cells. These escape mechanisms necessitate continued innovation in ACT to ensure durable and robust responses [124]. T-cell persistence, a critical determinant of long-term efficacy, is controlled by intrinsic cellular networks, including memory formation and metabolic fitness [125]. Additionally, nano-engineering and non-viral delivery systems are emerging to optimize ACT delivery and tumor penetration [ 126, 127] . Overall, ACT is a transformative approach in immunotherapy with diverse mechanisms tailored to improve efficacy and overcome resistance across various disease contexts [128] ( Table 2).

**Table:** **Table 2** Commercialized ACT products

Drug	Target	Type	Tumor	Approval	Ref.
Tisagenlecleucel	CD19	CAR-T	Acute lymphoblastic leukemia, B-cell lymphoma, follicular lymphoma	August 2017	[129]
Axicabtagene ciloleucel	CD19	CAR-T	Large B-cell lymphoma	October 2017	[129]
Lisocabtagene maraleucel	CD19	CAR-T	B-cell malignancy	May 2024	[130]
Idecabtagene vicleucel	BCMA	CAR-T	B-cell malignancy	March 2021	[130]
Lifileucel	Tumor-associated neoantigens	TIL	Advanced melanoma	February 2024	[131]
Afamitresgene autoleucel	MAGE-A4	TCR	Advanced synovial sarcoma	August 2024	[132]

ACT faces critical challenges, including limited persistence of transferred cells, immune-related toxicity, TME suppression, antigen escape, and suboptimal trafficking of therapeutic cells to tumor sites [120]. In the phase 3 ACT trial, axicabtagene ciloleucel (axi-cel), a CD19-directed CAR-T-cell therapy, demonstrated an 82% objective response rate and a 54% complete response rate in patients with refractory large B-cell lymphoma, with durable responses and a manageable safety profile despite notable adverse effects such as cytokine release syndrome and neurologic events [133]. In relapsed hematologic cancer, myeloablation and GVHD have strong toxic effects, which strongly undermines the effect of CAR-T-cell therapy [ 134, 135] . Moreover, inconsistencies in patient response highlight the need for more personalized and dynamic treatment strategies. The integration of circadian rhythm biology, a system that orchestrates daily cycles in physiological processes, offers a novel angle to enhance ACT outcomes. As challenges in CAR-T-cell therapy, such as cancer resistance, relapse, and toxicity, such as cytokine release syndrome (CRS) and neurotoxicity, are linked to the complex dynamics of CAR-T cells and their surroundings, the identification of predictive biomarkers, including core clock genes for therapeutic response and toxicity, as well as insights into CAR-T-cell exhaustion, persistence, and clonal expansion post-infusion overcomes the current limitations [136]. The circadian clock tightly regulates immune cell trafficking, differentiation, and functional states, suggesting that timing ACT administration in sync with these cycles could improve efficacy and reduce toxicity [5]. For example, circadian timing affects T-cell homing to lymphoid tissues, as demonstrated in adoptive transfer models showing differential outcomes depending on the circadian phase of cell infusion [58]. This synchronization could enhance cell engraftment and anti-tumor activity. Furthermore, circadian modulation of immune checkpoints and cytokine signaling pathways may reduce immune-related adverse events [106]. In brain cancers, circadian-based scheduling of immunotherapy has improved the precision and therapeutic index, suggesting broader applications of “chrono-immunotherapy” in ACT [114]. A study revealed that cell cycle arrest and apoptosis are induced in glioblastomas following the inhibition of BMAL1 or CLOCK in glioblastomas, revealing the potential of combination therapy with BMAL1-CLOCK negative regulators and ACT [137]. Such approaches may evolve into a new paradigm termed “circadian precision medicine,” which aligns therapy with patients’ biological rhythms to enhance efficacy and minimize harm [138]. In summary, harnessing the circadian rhythm may help overcome major obstacles in ACT by optimizing cell function, timing interventions to improve immune readiness, and enabling personalized chronotherapy regimens. Future trials should incorporate circadian biology into ACT protocols for maximized benefit. Immune cells, including T cells and DCs, exhibit circadian-regulated behaviors in trafficking, antigen presentation, and cytokine production. DC-dependent T-cell priming involves circadian oscillations regulated by core clock genes, such as *BMAL1* and *CLOCK*, that modulate antigen presentation efficiency and co-stimulatory molecule expression [5]. This rhythmic regulation influences the efficiency of T cell responses, which is critical in ACT strategies, where the timing of infusion or activation influences therapeutic outcomes. Recent work has suggested that the TME itself, including endothelial cells, has circadian properties that affect CD8 ^+^ T-cell infiltration and function, directly modulating immunotherapy outcomes [82]. Targeting these temporal windows has been proposed as a means to synchronize treatment administration for maximal impact. Furthermore, in brain cancer immunotherapy, integrating circadian-informed timing with ACT and checkpoint blockade has shown promise in preclinical models, suggesting that this synergy may enhance therapeutic precision [114]. Mechanistically, circadian rhythms also regulate hematopoietic stem and progenitor cells (HSPCs), from which immune cells are derived. The timing of stem cell differentiation is influenced by light cycles and endogenous clocks, which could affect the replenishment and function of immune effectors used in ACT [139]. These insights suggest that leveraging circadian biology through administration timing or gene-editing strategies targeting clock genes could optimize ACT efficacy. Despite the growing evidence base, clinical translation requires further studies, particularly in aligning the patient circadian phase with therapy scheduling.

### RORγ agonists promote therapeutic effects

The nuclear receptor RORγ, a core circadian regulator, enhances antitumor immunity through direct transcriptional control of CD8 ^+^ T cell effector differentiation and metabolic reprogramming. Agonists of RORγ promote the differentiation of Tc17 cells, a subtype of CD8 ^+^ T cells, and increase the expression of effector molecules such as IFN-γ and granzyme B, contributing to improved tumor-killing capacity [92]. Notably, RORγ agonists also suppress inhibitory receptors such as PD-1 on T cells, thereby rescuing their function within the TME [140]. Mechanistically, RORγ agonists such as LYC-54143 and LYC-55716 (cintirorgon) have been shown to promote CD8 ^+^ T cell infiltration and cytotoxic activity in tumors [ 141, 142] . In addition, RORγ agonists enhance memory and stem-like phenotypes in T cells, which contribute to durable responses [140]. Research has also shown that RORγ agonists induce CXCL10 via monocyte-derived DCs, which facilitates immune cell recruitment and synergizes with ICB [141]. When combined with PD-1 inhibitors, RORγ agonists have synergistic effects, including further downregulation of PD-1 expression, potentiating checkpoint blockade efficacy [143]; greater tumor growth inhibition; improved survival outcomes in preclinical models [144]; and amplified Th17 responses and enhanced CD8 ^+^ T-cell priming, particularly when additional co-stimulatory molecules such as CD27 agonists are used [145]. In essence, the combination of chronotherapy with ICB orchestrates a robust immune activation strategy that reshapes the TME, reprograms T cells for cytotoxicity and memory, and substantially improves therapeutic responses in cancer models.

## Conclusion

The intricate interplay between circadian rhythms and immunotherapy represents a groundbreaking frontier in cancer treatment, offering profound implications for optimizing therapeutic efficacy and patient outcomes. Circadian transcriptional networks, centered on BMAL1/CLOCK activity, dynamically regulate immune homeostasis by timing lymphocyte trafficking, cytokine secretion peaks, and antigen presentation capacity. These rhythms not only are pivotal for maintaining homeostasis but also orchestrate dynamic cellular crosstalk within the neoplastic milieu and modulate responses to immunotherapy. Contemporary research has established circadian regulation as a fundamental determinant of immune cell dynamics, influencing different aspects of antitumor immune responses, from lymphocyte trafficking to phagocytic activity. Key immune effectors, including T lymphocytes, natural killer cells, and monocyte-derived macrophages, demonstrate cell-autonomous circadian regulation of their functional programs, exhibiting time-dependent oscillations in tumor infiltration capacity, cytotoxic granule production, and inflammatory mediator secretion. Disruptions to these rhythms can lead to immunosuppressive TMEs, characterized by increased numbers of MDSCs and TAMs. Conversely, aligning immunotherapy administration with peak immune activity and chronotherapy can increase T-cell activation, improve tumor surveillance, and reduce adverse effects. Clinical evidence suggests that timing ICB therapies to coincide with circadian peaks in immune function may improve response rates and survival outcomes, underscoring the importance of circadian-informed treatment protocols.

The identification of the molecular mechanisms underlying circadian regulation of the TME further highlights the potential for therapeutic innovation. Core clock genes such as BMAL1 and PER2 act as tumor suppressors, and their dysregulation is linked to aggressive cancer phenotypes and poor prognosis. BMAL1, for example, modulates TGF-β signaling and stromal remodeling, whereas PER2 influences DNA repair and apoptosis. Targeting these pathways, either through pharmacological agents or gene-editing technologies, could restore circadian integrity or counteract tumor progression. Additionally, RORγ agonists have emerged as promising tools to amplify anti-tumor immunity by enhancing CD8 ^+^ T-cell function and synergizing with PD-1 inhibitors. These findings pave the way for combination therapies that integrate circadian modulation with existing immunotherapies.

**Figure 2:**
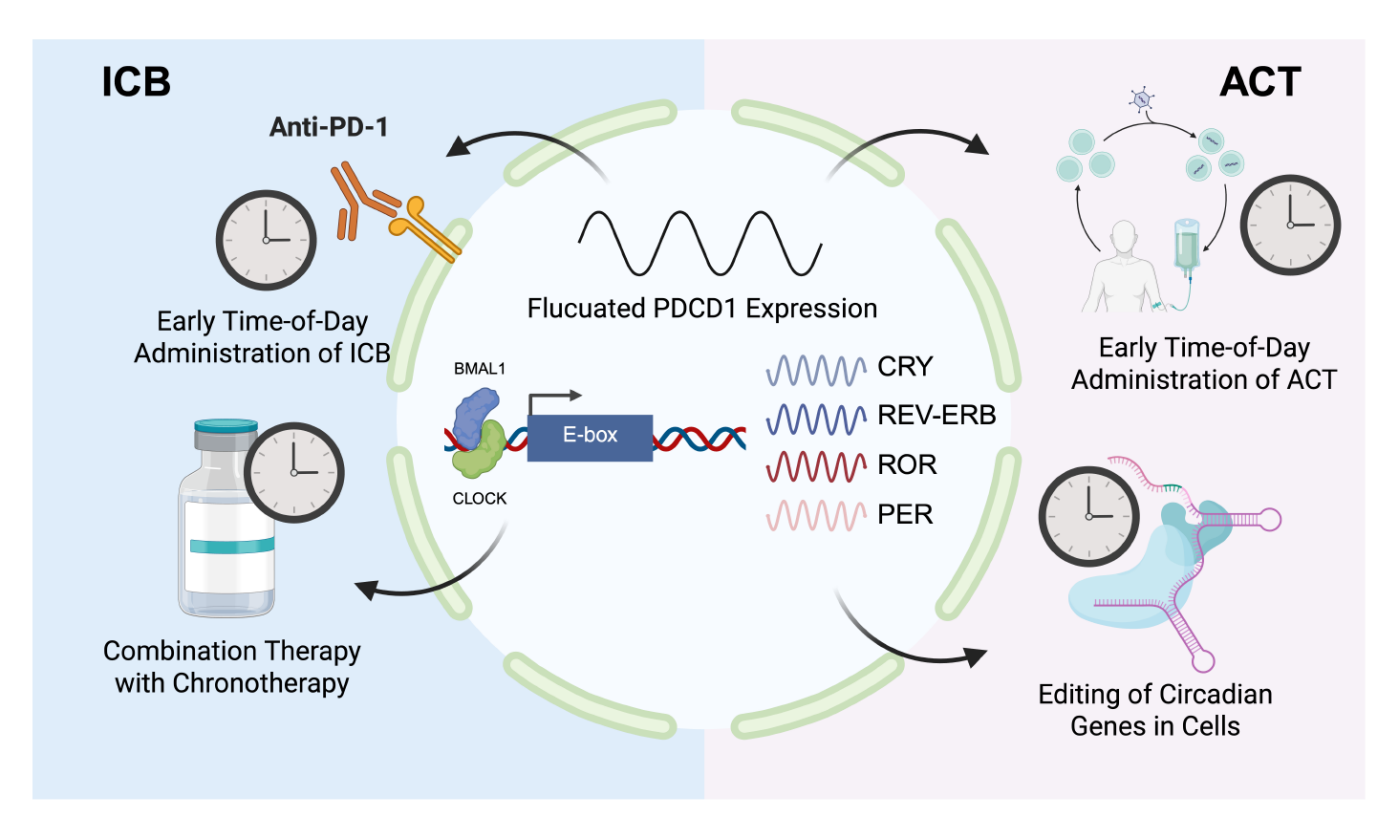
Figure 2 How circadian rhythm improves the therapeutic effect of ICB and ACT The expression of PDCD1 fluctuates periodically, affecting the treatment effect of ICB and ACT. The circadian genes BMAL1 and CLOCK form a heterodimer that combines the E box promoter elements to regulate the transcription levels of downstream circadian genes, including CRY,REV-ERB,ROR, and PER.

Despite these advancements, challenges remain in translating circadian biology into clinical practice. Heterogeneity in patient circadian rhythms, variability in tumor types, and the complexity of immune-TME interactions necessitate personalized approaches. Biomarkers such as circadian gene signatures and immune cell infiltration patterns could help identify patients most likely to benefit from chronotherapy. Moreover, lifestyle interventions, including sleep hygiene and light therapy, may complement pharmacological treatments to restore circadian health and improve immunotherapy outcomes. The strategic timing of immunotherapies according to circadian principles constitutes a breakthrough in oncology practice, potentially increasing response rates while minimizing immune-related adverse events. By leveraging the temporal dynamics of immune function and TME regulation, researchers and clinicians can develop more precise, effective, and less toxic treatment strategies. Future directions should focus on large-scale clinical trials to validate circadian-based protocols, explore novel circadian targets, and refine personalized chronotherapy regimens. As the understanding of chronoimmunology has deepened, the potential to revolutionize cancer care and improve patient survival has increased ( Figure 2). The synergy between circadian biology and immunotherapy not only illuminates new pathways for scientific discovery but also offers hope for more successful and sustainable cancer treatments, underscoring the need for further research to translate these findings into clinical practice and develop personalized treatments.
